# Influence of MicroRNA‐141 on Inhibition of the Proliferation of Bone Marrow Mesenchymal Stem Cells in Steroid‐Induced Osteonecrosis *via* SOX11

**DOI:** 10.1111/os.12603

**Published:** 2020-01-08

**Authors:** Chen‐yang Meng, Fei Xue, Zhen‐qun Zhao, Ting Hao, Shi‐bing Guo, Wei Feng

**Affiliations:** ^1^ Orthopedics Department Second Affiliated Hospital of Inner Mongolia Medical University Hohhot China; ^2^ Orthopedics Department Inner Mongolia Institute of Orthopedics Hohhot China

**Keywords:** Bone marrow mesenchymal stem cells, Glucocorticoid, MicroRNA‐141, SOX11, Steroid induced avascular necrosis of femoral head

## Abstract

**Objective:**

To investigate whether miR‐141 and the sex determination region of Y chromosome box 11 (SOX11) play roles in steroid‐induced avascular necrosis of the femoral head (SANFH), and to explore whether miR‐141 could target SOX11 to influence the proliferation of bone marrow mesenchymal stem cells (BMSC).

**Methods:**

Bone marrow mesenchymal stem cells (BMSC) were isolated and cultured from 4‐week‐old Sprague Dawley rats. A flow cytometry assay was performed to identify BMSC. BMSC were divided into two groups: a control group and a dexamethasone (DEX) group. BMSC were transfected by miR‐141 mimic, miR‐141 inhibitor, and SOX11. Real‐time polymerase chain reaction (PCR) assay was performed to investigate the mRNA expression of miR‐141 and SOX11. The results were used to determine the effect of transfection and to verify the expression in each group and the association between miR‐141 and SOX11. Luciferase reporter assay revealed the targeted binding site between miR‐141 and the 3′‐untranslated region of SOX11 mRNA. MTT assays were performed to investigate the proliferation of BMSC in the miR‐141 mimic, miR‐141 inhibitor, and SOX11 groups.

**Result:**

The results of the flow cytometry assay suggested that cells were positive for CD29 and CD90 while negative for CD45. This meant that the isolated and cultured cells were not hematopoietic stem cells. In addition, cell transfection was successful based on the expression of miR‐141 and SOX11. According to the results of real‐time PCR assay, the mRNA expression of miR‐141 in SANFH was upregulated (4.117 ± 0.042 *vs* 1 ± 0.027, *P* < 0.001), while SOX11 was downregulated (0.611 ± 0.055 *vs* 1 ± 0.027, *P* < 0.001) compared with the control group. Based on the results of the luciferase experiment, MiR‐141 could directly target the expression of SOX11. Inhibition of miR‐141 could upregulate the expression of SOX11 (2.623 ± 0.220 *vs* 1 ± 0.095, *P* < 0.001) according to the results of a real‐time PCR assay. MiR‐141 inhibited the proliferation of BMSC (0.618 ± 0.092 *vs* 1.004 ± 0.082, *P* < 0.001), while suppression of miR‐141 increased the proliferation of BMSC (0.960 ± 0.095 *vs* 0.742 ± 0.091, *P* < 0.001). Furthermore, according to the results of the MTT assay, SOX11 promoted the proliferation of BMSC (1.064 ± 0.093 *vs* 0.747 ± 0.090, *P* < 0.001).

**Conclusion:**

MiR‐141 inhibited the proliferation of BMSC in SANFH by targeting SOX11. Inhibition of miR‐141 upregulated the expression of SOX11 and promoted the proliferation of BMSC. MiR‐141 and SOX11 could be new targets for investigating the mechanism of SANFH.

## Introduction

Glucocorticoids are used in the treatment of many inflammatory, allergic, immunologic, and malignant disorders. Steroid‐induced avascular necrosis of the femoral head (SANFH) can be induced by the long‐term use of glucocorticoids^1^, ^2^. There are 20 million necrosis of femoral head patients in the world and more than 5–7.5 million in China^3^. SANFH is a great physical and psychological burden on patients. Thus, it is urgent to understand the pathogenesis and provide corresponding treatment strategies. However, the mechanism of SANFH remains unclear. It is well known that long‐term use of glucocorticoids leads to abnormal lipid distribution. One possible mechanism for SANFH involves alterations in circulating lipids with resultant microemboli in the arteries supplying bone^4^. Another is the increasing bone marrow adipocyte size and number, which may be able to block the venous outflow^5^. In addition, apoptosis and autophagy of osteocytes were induced by using glucocorticoids^6^, ^7^. Cell death could be caused by ischemia and hypoxia. The death of osteocytes could lead to a decrease in bone mass, further increasing the risk of trabecular fracture^7^. In addition, glucocorticoids have multiple effects on bone formation^8^. Several studies argue that increased sclerostin production through osteocytes could inhibit long‐term bone formation, resulting in suppression of the Wnt/β‐catenin pathway and an increase of the peroxisome proliferator‐activated receptor. Finally, pluripotent precursor cells tend to differentiate into adipocytes rather than osteoblasts^9^, ^10^. It is thus clear that improving the treatment options for lipid metabolism disorder and increasing the bone mass are crucial for prevention of SANFH.

The identification of a population of cells in the bone marrow that could form a number of mesenchymal cell populations *ex vivo* led to the concept of a mesenchymal stem cell (MSC)^11^. Matsuya et al. (2008) suggested that the injection of autologous MSC into the femur could alleviate the progression of disease in steroid‐induced osteonecrosis patients treated with high‐dose short‐term steroids, which revealed that MSC had a positive influence on SANFH^12^. It has been verified that BMSC could be progenitor cells that differentiate into osteoblasts, osteocytes, adipocytes, and chondrocytes^13^, ^14^. In addition, they are reported to be closely associated with lipid metabolism and osteogenic differentiation^15^. The proliferation and differentiation of BMSC are reported to be associated with osteopenia‐related disease, including osteoporosis and steroid‐induced osteonecrosis^16^, ^17^. This might be due in part to the combination of increasing of bone resorption and suppression of bone formation. Studies have shown that glucocorticoids could suppress bone formation through a direct effect on BMSC^18^. In addition, use of glucocorticoids leads to osteoporosis, which further causes fractures of the trabecular and delays in bone repair so that collapse of the femoral head can, finally, occur^19^. Lee *et al*. (2006) report that BMSC are impaired in steroid‐induced osteonecrosis due to a decrease in their proliferative ability^20^. Based on the above, investigating the regulators of BMSC in proliferation and differentiation might provide new insight into the mechanism of SANFH.

Victor Ambros first discovered microRNA (miRNA) and revealed that miRNA could regulate more than 30% of human genes; in addition, they are regarded as endogenous small RNA molecules with biologically regulatory functions^21^, ^22^. MiRNA is a non‐coding single‐stranded small RNA molecule that promotes degradation of target genes by binding to their 3′‐UTR region^23^. Furthermore, miRNA have regulatory roles in gene expression at the post‐transcriptional level^24^. Sangani et al. (2015) argue that miRNA are involved in the regulation of cell functions, including differentiation, proliferation, and migration of BMSC^25^. In addition, miRNA are associated with glucocorticoid‐related pathological processes^26^, ^27^. MircroRNA‐141 (miR‐141) was reported to be associated with cell proliferation in breast cancer cells^28^. Research has reported that miR‐141 decreasing osteogenic and miRNA inhibitors could increase the osteogenic gene expression in BMSC^29^. The SOX (sex determination region of Y chromosome box) family is a group of transcription factors that play a key role in cell growth^30^. It has been found that overexpression of SOX11 (sex determination region of Y chromosome box 11) could inhibit the proliferation of ovarian cancer, while downregulation of SOX11 could restrain the proliferation of thyroid tumors^31^, ^32^. In addition, it has been reported that blocked endogenous glucocorticoid receptors could increase the proliferation of BMSC and upregulate expression of osteogenic and 11 (SOX11)^33^. SOX11 was also found to be associated with capability of osteochondral differentiation of BMSC^34^. MiR‐141 and SOX11 were both associated with proliferation and osteogenic differentiation of BMSC; the correlation between them is still unclear.

From the above, miR‐141 and SOX11 could be potential regulators in BMSC and further affect proliferation and differentiation of BMSC to prevent or delay the progression of SANFH. However, the roles of miR‐141 and SOX11 in SANFH have been studied little and the correlation between miR‐141 and SOX11 remains unclear. Further exploration is necessary.

To solve these issues, in our study, we investigated the regulation of miR‐141 and SOX11 in SANFH. Furthermore, we explore the association between miR‐141 and SOX11 and whether miR‐141 and SOX11 can regulate the proliferation of BMSC. Finally, we verify whether inhibition of miR‐141 can target SOX11 to promote the proliferation of BMMSC.

## Materials and Methods

### *Cell Culture and Transfection*

Three 4‐week‐old male Sprague Dawley rats (SCXK2017‐0001) were killed by cervical dislocation method. The weights of the rats were 69.3, 70.1, and 69.5 g, respectively. The bilateral femurs and tibias were removed under sterile conditions and the attached muscles were removed. A 10‐mL syringe was used to rush the bone marrow cells from femurs and tibias. This was repeated several times, until the femur and tibia appear pale. Cells suspensions were collected. After removing impurities and centrifuging, the α‐MEM (Hyclone, USA) was added to the mix. The cells were incubated at 37°C with 5% CO_2_. The culture medium was removed after cells grew to 80%–90%. PBS (Hyclone, USA) was used to wash the cells. After trypsin (Hyclone, USA) digestion and centrifugation, the cells were subcultured at 1:3.

The BMSC were divided into two groups. The group control was the normal BMSC and the group dexamethasone (DEX) was BMSC affected by dexamethasone with the final concentration at 1 μmol/L. The mRNA analysis was performed after incubation for 24 h.

Negative control (NC), miRNA mimics of miR‐141, miRNA inhibitors and overexpression vector of SOX11 were purchased from Shanghai Gemma Pharmaceutical (Shanghai, China). Lipofectamine 3000 (Invitrogen Life Technologies, Carlsbad, CA, USA) was used for transfecting BMSC according to the manufacturer's instructions. Transfected cells were used for mRNA analysis and the cell proliferation assay.

### *Flow Cytometry Assay*

Bone marrow mesenchymal stem cells were identified by flow cytometry assay. The cells were washed with PBS. The cells were then resuspended in the PBS‐2%BSA. The cells were adjusted to 2 × 10^7^ cells/mL. Then we added the primary antibody including CD29 Monoclonal Antibody (eBioscience, 11–0291‐82, USA), CD29 Monoclonal Antibody (eBioscience, 11–0461‐82, USA), and CD90 Monoclonal Antibody (eBioscience, MA1‐80648, USA) at the recommended concentration according to the manufacturer's instructions. After incubation and centrifugation, flow cytometry assay (Becton Dickinson, USA) was performed.

### *Cell Proliferation Assay*

Bone marrow mesenchymal stem cells (5000/per well) were plated in a 96‐well plate. Each well had 3000 cells. After the cells were attached to the well, three subgroups including the SOX11 group and the control group, the miR‐141 inhibitor group and the control group, and the miR‐141 mimic group and the control group were divided. Each subgroup was set the five same wells. Cells were placed in an incubator for 72 h. Then 10 μL of methyl thiazolyl tetrazolium (Beyotime, China) was added in each well, and we cultivated the plates for 3 h in the cell culture incubator. Finally, we added approximately 150 μL of DMSO (Sigma, USA) to each well after removing the medium. An Absorbance Microplate Reader (Thermo fisher, Multiskan 51119000, USA) platform was used to measure the absorbance at 570 nm.

### *Luciferase Experiment*

We polymerase chain reaction (PCR)‐amplified 3′‐UTR of SOX11 and cloned this into a pmirGLO Vector to construct the reporter assay vector. We transfected 60% confluent cells of 60% in 24‐well plates with reporter vector using 5 μL Lipofectamine 3000 and 40 ng pRL‐Tk. BMSC were co‐transfected with the constructed wild‐type or mutant pmirGLO Vector (100 ng), and either 100 ng of NC or miR‐ mimic per well, respectively. We isolated cellular extracts at 48‐h post‐transfection, and evaluated luciferase activity with the help of the Dual Luciferase Reporter Assay System (Promega, USA).

### *Real‐time Polymerase Chain Reaction Assay*

We used TRIzol Reagent (Invitrogen, USA) to isolate total RNA from cells according to the instructions of the manufacturer. The PrimeScript RT Reagent Kit (TaKaRa, Dalian, China) was used for conversion of RNA into cDNA; the miRNA Extraction Kit (Ribobio, Guangzhou, China) helped to achieve the same objective with respect to miRNA. Quantification of transcripts was attained by performing RT‐PCR using SYBR Premix Ex Taq (TaKaRa, Dalian, China). We used the ABI StepOne Plus Real‐Time PCR System (Applied Biosystems; Thermo Fisher Scientific,USA) to perform quantitative PCR (qPCR) using the SYBR‐Green PCR Kit (TaKaRa, Dalian, China). The qPCR protocol consists of the following steps: (i) initial denaturation for 5 min at 95°C; and (ii) 40 cycles involving denaturation at a temperature of 95°C for 10 s, followed by annealing and extension at 60°C for 34 s. We repeated the experiments five times.

The mRNA expressions of miR‐141 and SOX11 were measured in control and SANFH groups. The mRNA expressions of miR‐141 and SOX11 were measured in the group which was transfected by miR‐141 mimic and miR‐141 inhibitor.

### *Data Collection*

#### *Flow Cytometry Assay*

The expression of the cell markers, including CD29, CD45, and CD90 specific for mesenchymal and hematopoieticstem cells, was analyzed with flow cytometry. According to the results of the flow cytometry assay, it could be identified whether BMSC‐positive markers CD29 and CD90 but not the marker CD45 were on the surface of the cells. Then, we could identify whether or not the cells we cultured were hematopoietic stem cells.

#### *MRNA Analysis*

For mRNA analysis, real‐time PCR assays were performed. The relative quantity (RQ) value was used to indicate the expression level of mRNA. Application of double Δ Δ CT analysis was used to calculate the RQ value. The values we obtained could be used to evaluate the expression of mRNA.

#### *Proliferation Assay*

For cell proliferation assay, an Absorbance Microplate Reader platform was used to measure the absorbance at 570 nm. The measured optical density (OD) values were used to indicate the cell proliferation ability.

#### *Luciferase Activity*

With the help of the Dual Luciferase Reporter Assay System (Promega, USA), we could evaluate luciferase activity values. The values were used to verify whether miR‐141 could directly target SOX11‐3′‐UTR.

### *Statistical Analysis*

The results were represented in the form of mean standard deviation. We used SPSS 17.0 software (IBM, Armonk, NY, USA) for statistical analysis. We performed *t*‐tests to compare the differences between the experimental group and the control group. The normality test was performed first and then an independent samples *t*‐test was performed to compare differences because the data followed a normal distribution. A *P*‐value <0.05 was deemed statistically significant.

## Result

### *Cell Identification of Bone Marrow Mesenchymal Stem Cells*

**Figure 1:**
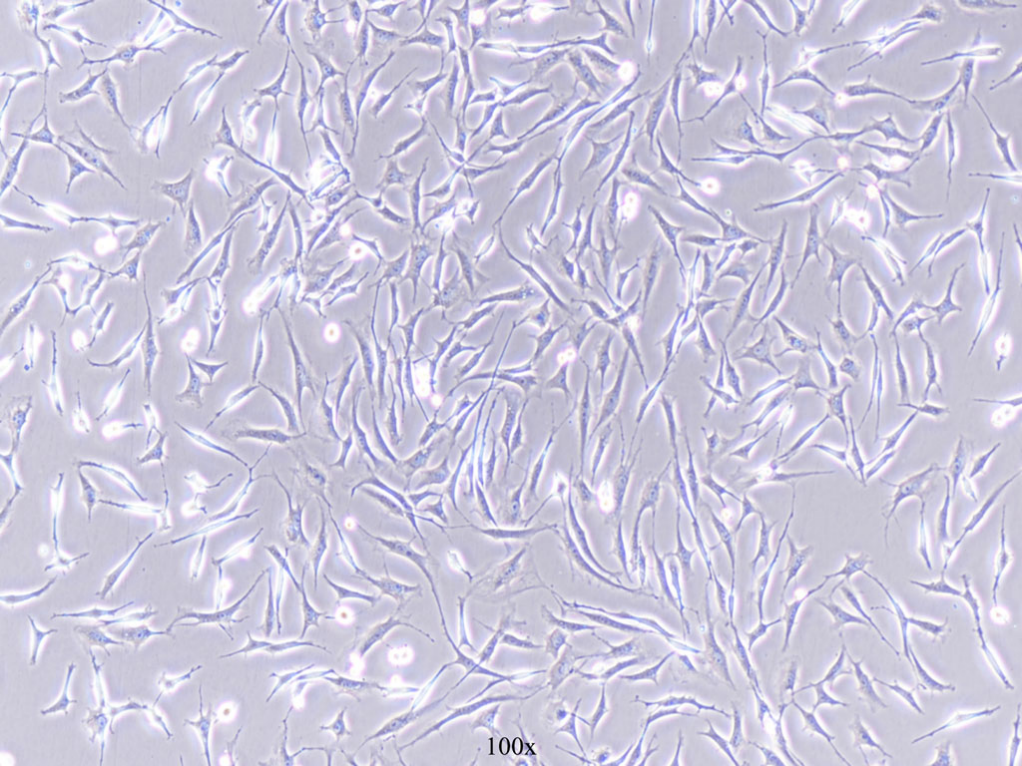
The bone marrow mesenchymal stem cells (BMSC) under 100× optical microscope (black arrow).

**Figure 2:**
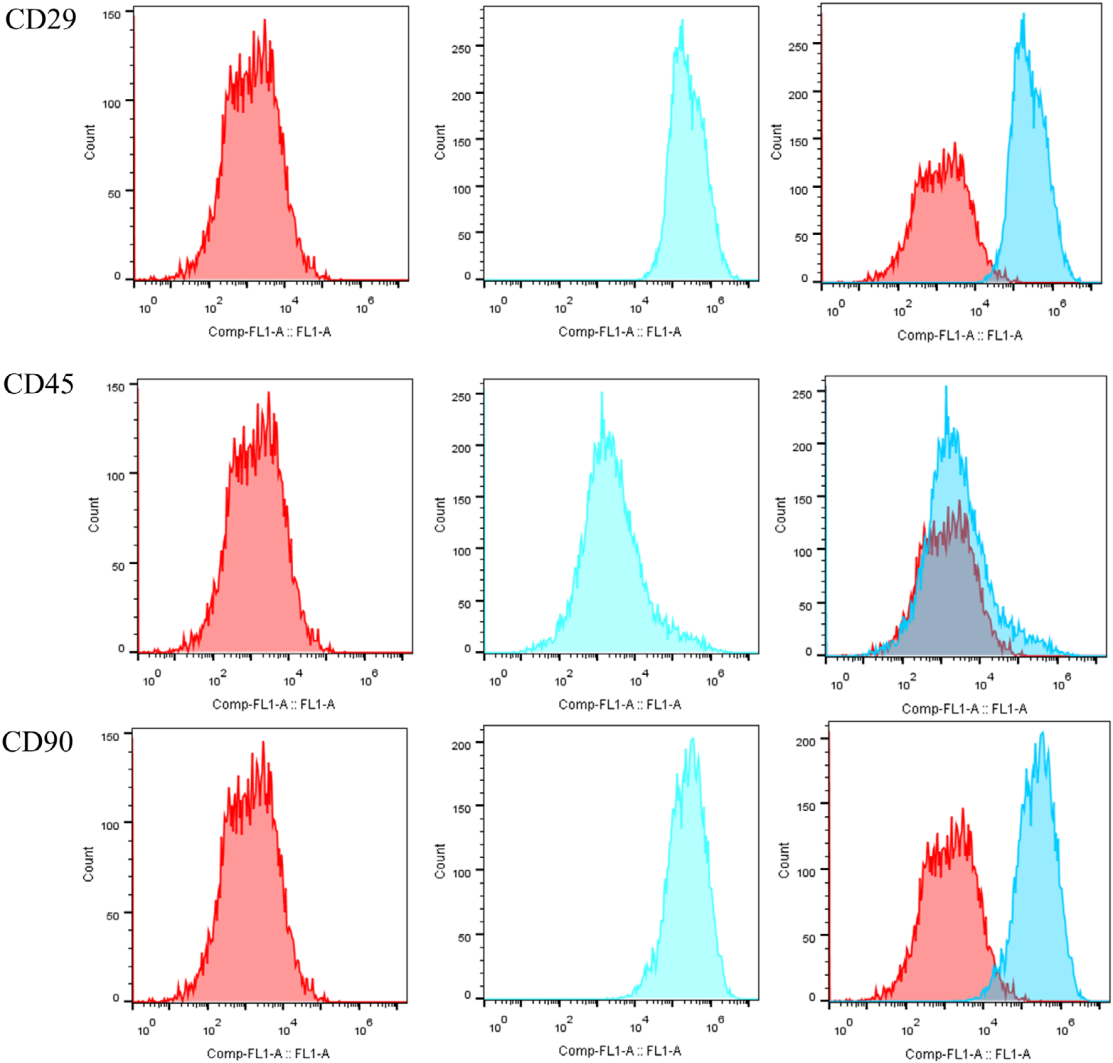
The flow cytometry assay was used to identify bone marrow mesenchymal stem cells (BMSC). The red histogram shows the situation before staining and the blue histogram shows cells following staining by anti‐rat CD29, CD45, and CD90 fluorescein isothiocyanate. The cells were positive for CD29 and CD90 while negative for CD45. BMSC, bone marrow mesenchymal stem cells; CD, cluster of differentiation.

Fig. 1 shows the morphology of BMSC under an ordinary optics microscope. The results of the flow cytometry assay showed that the cells were positive for CD29 and CD90, while the cells were negative for CD45 (Fig. 2).

### *Cell Transfection of Bone Marrow Mesenchymal Stem Cells*

**Figure 3:**
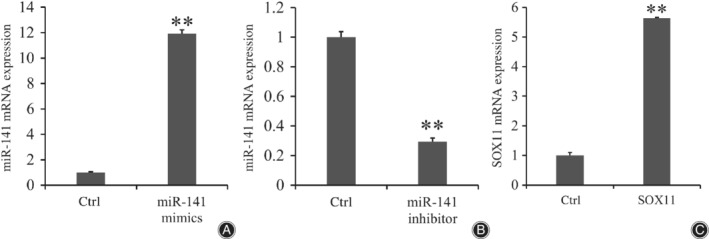
The results of transfection by miR‐141 mimic (A) miR‐141 inhibitor (B) and SOX11 (C). The mRNA expression in miR‐141 mimic and SOX11 groups was higher than for each control group (*P* < 0.01), while the mRNA expression in the miR‐141 inhibitor group was lower than for each control group (*P* < 0.01). The results showed that transfections were successful. Application of double Δ Δ CT analysis was used to calculate the RQ value. Data is presented as RQ value ± standard deviation. **P < 0.01; * means compared with control group. CT, cycle threshold; Ctrl, control group; RQ, relative quantity.

The BMSC were transfected by miR‐141 mimic, miR‐141 inhibitor, and SOX11. The mRNA expression of miR‐141 in the miR‐141 transfection group was significantly higher than that of the control group and the average expression value of the miR‐141 mimic transfection group was nearly 11.9 times that of the control group (11.918 ± 0.313 *vs* 1 ± 0.043, *P* < 0.001; Fig. 3A). The mRNA expression of miR‐141 in the miR‐141 inhibitor transfection group was significantly lower than that of the control group and the average expression value of the control group was nearly 3.4 times that of the miR‐141 inhibitor transfection group (1 ± 0.010 *vs* 0.296 ± 0.023, *P* < 0.001; Fig. 3B). The mRNA expression of SOX11 in the SOX11 transfection group was significantly higher than that of the control group and the average expression value of the SOX11 transfection group was nearly 5.6 times that of control group (5.638 ± 0.023 *vs* 1 ± 0.037, *P* < 0.001; Fig. 3C).

### *The mRNA Expression of miR‐141 in Dexamethasone*

**Figure 4:**
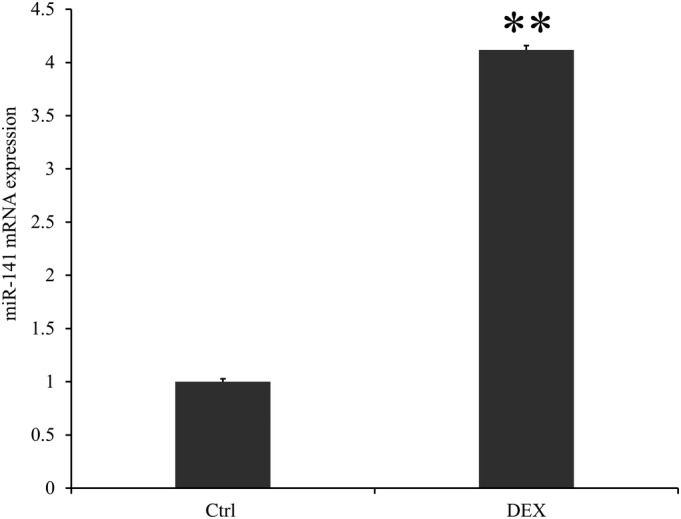
The mRNA expression of miR‐141 in control and DEX groups. Control was the group without treatment. DEX was the group treated by dexamethasone for 24 h. The mRNA expression of miR‐141 was higher in the DEX group compared with the control group (*P* < 0.01), which meant that DEX could promote the expression of miR‐141. **P < 0.01, * means compared with control group. Application of double Δ Δ CT analysis to calculate the RQ value. Data is presented as RQ value ± standard deviation. **P < 0.01; * means compared with control group. CT, cycle threshold; Ctrl, control group; DEX, dexamethasone; RQ, relative quantity.

The mRNA expression of miR‐141 in the DEX group was significantly higher compared with the control group and the average expression value of miR‐141 in the DEX group was nearly 4.1 times that of the control group (4.117 ± 0.042 *vs* 1 ± 0.027, *P* < 0.001; Fig. 4).

### *mRNA Expression of SOX11 in Dexamethasone*

**Figure 5:**
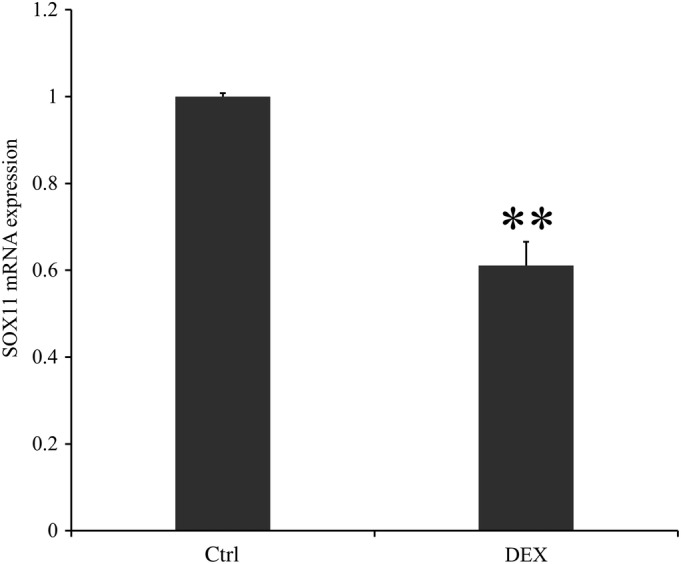
The mRNA expression of SOX11 in control and DEX. Control was the group without treatment. DEX was the group treated by dexamethasone for 24 h. The mRNA expression of SOX11 was lower in the DEX group compared with the control group (*P* < 0.01), which meant that DEX could inhibit the expression of SOX11. **P < 0.01; * means compared with the control group. Application of double Δ Δ CT analysis to calculate the RQ value. Data is presented as RQ value ± standard deviation. **P < 0.01; * means compared with control group. CT, cycle threshold; Ctrl, control group; DEX, dexamethasone; RQ, relative quantity.

The mRNA expression of SOX11 in the DEX group was significantly lower than that of the control group and the average expression value of SOX11 in the control group was nearly 1.6 times that of the DEX group (1 ± 0.008 *vs* 0.611 ± 0.055, *P* < 0.001; Fig. 5).

### *Association Between miR‐141 and SOX11*

**Figure 6:**
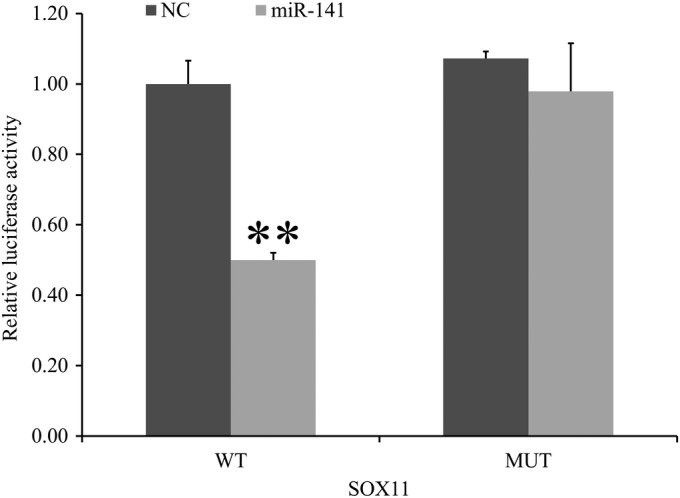
The relative fluorescence unit values of BMSC was assessed after co‐transfection of miR‐141 and SOX11 wild‐type plasmids or mutant plasmids. There was statistical difference between the wild‐type group and the negative control group (*P <* 0.01), while no statistical difference was found between the mutant type group and the negative control group (*P* > 0.05). The results suggested that miR‐141 could directly target SOX11‐3′‐UTR. **P < 0.01; * means compared with NC group. BMSC, bone marrow mesenchymal stem cells; MUT, mutant type; NC, negative control; WT, wild‐type.

**Figure 7:**
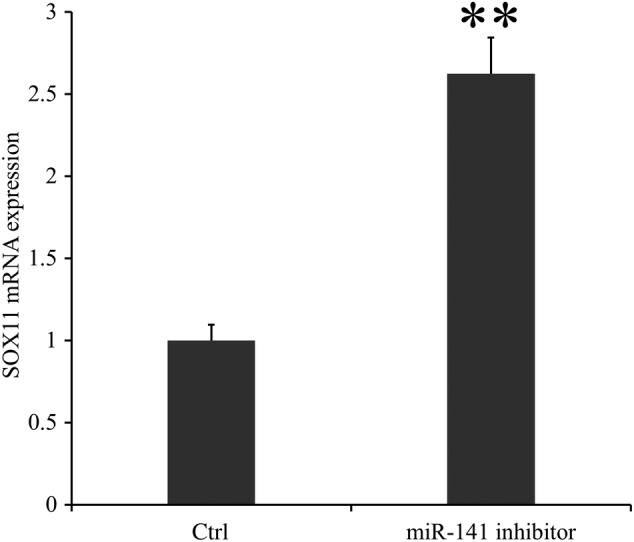
The SOX11 mRNA expression in group control and miR‐141 inhibitor. In the group miR‐141 inhibitor, the BMSC were transfected by miR‐141 inhibitor. It was found that the mRNA expression of SOX11 was higher in the miR‐141 inhibitor group compared with the control group (*P* < 0.01), which meant that SOX11 could be upregulated by inhibiting miR‐141.Application of double Δ Δ CT analysis to calculate the RQ value. Data is presented as RQ value ± standard deviation. **P < 0.01. * means compared with the control group. BMSC, bone marrow mesenchymal stem cells; CT, cycle threshold; Ctrl, control group; RQ, relative quantity.

The luciferase experiment was performed to assess whether miR‐141 was directly targeting SOX11 expression through the target site in the 3′‐UTR of SOX11. The average value of luciferase activity in the wild‐type group was significantly lower than that of the negative control group and the average luciferase activity value in the negative control group was nearly two times that of the wild‐type group (1 ± 0.070 *vs* 0.500 ± 0.020, *P* < 0.001), while no statistical difference was found between the mutant type group and the negative control group (0.980 ± 0.140 *vs* 1 ± 0.020, *P* > 0.05; Fig. 6). According to the results of the real‐time PCR assay, the mRNA expression of SOX11 in the miR‐141 inhibitor transfection group was significantly higher than that of the control group and the mRNA average expression value of SOX11 in the miR‐141 inhibitor transfection group was nearly 2.6 times that of the control group (2.623 ± 0.220 *vs* 1 ± 0.095, *P* < 0.001; Fig. 7).

### *Proliferation of Bone Marrow Mesenchymal Stem Cells Affected by miR‐141*

**Figure 8:**
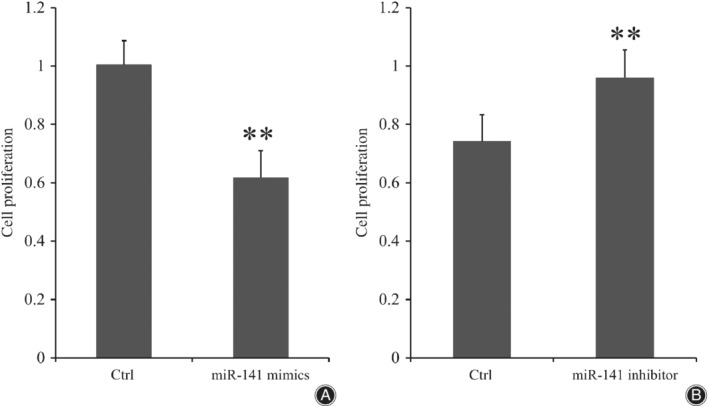
The cell proliferation ability of BMSC, which were transfected by miR‐141 (A) or miR‐141 inhibitor (B). The OD values were higher in the miR‐141 inhibitor group compared with the control group (*P* < 0.01) and OD values were lower in the miR‐141mimics group compared with control group, which meant that the cell proliferation ability of BMSC could be inhibited by miR‐141 while inhibition of miR‐141 could enhance the proliferation ability of BMSC (*P* < 0.01). Data is presented as OD value ± standard deviation. **P < 0.01; * means compared with control group. BMSC, bone marrow mesenchymal stem cells; Ctrl, control group; OD, optical density.

It was showed in Fig. 8A that the average OD value in the miR‐141 mimic transfection group was significantly lower than that of the control group and the average OD value in the control group was nearly 1.6 times that of the miR‐141 mimic transfection group (0.618 ± 0.092 *vs* 1.004 ± 0.082, *P <* 0.001). In contrast, the average OD value in the miR‐141 inhibitor transfection group was significantly higher than that of the control group and the average OD value in the miR‐141 inhibitor transfection group was nearly 1.3 times that of the control group (0.960 ± 0.095 *vs* 0.742 ± 0.091, *P* < 0.001; Fig. 8B).

### *Proliferation of Bone Marrow Mesenchymal Stem Cells Affected by SOX11*

**Figure 9:**
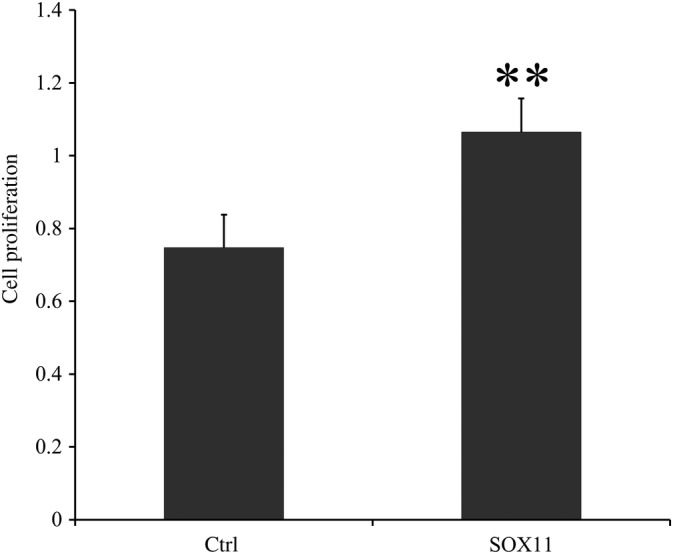
The cell proliferation ability of BMSC, which were transfected by SOX11. The OD values were higher in the SOX11 group compared with the control group (*P* < 0.01), which meant that the cell proliferation ability of BMSC could be enhanced by SOX11. Data is presented as OD value ± standard deviation. **P < 0.01; * means compared with control group. BMSC, bone marrow mesenchymal stem cells; Ctrl, control group; OD, optical density.

In Fig. 9, the average OD value in the SOX11 transfection group was significantly higher than that of the control group and the average OD value in the SOX11 transfection group was nearly 1.3 times that of the control group (1.064 ± 0.093 *vs* 0.747 ± 0.090, *P* < 0.001).

## Discussion

Treatment and prevention of SANFH remains a challenge for clinical medicine. It has been verified that glucocorticoids are associated with lipid metabolism. In addition, it was found that collapse of the trabecular bone and increasing empty lacunae occurred in SANFH^35^. The apoptosis and autophagy rates of osteocyte increased in the model of SANFH^36^. BMSC was found to be associated with lipid metabolism and osteogenic differentiation^13^, ^14^. Recent research revealed that osteogenic differentiation of BMSC in the SANFH was suppressed while lipid differentiation was increased^37^. These findings suggested that BMSC play a significant role in pathological changes associated with SANFH. It is crucial to investigate regulators of BMSC in SANFH. According to our results, we found that miR‐141 and SOX11 are potential regulatory factors of BMSC.

Our study revealed that miR‐141 could be a regulator of BMSC by inhibiting proliferation. It has been reported that miR‐141 reduced osteogenic differentiation in BMSC by regulating SVCT2^29^. MiR‐141 was reported to be associated with tissue repair induced by mesenchymal stromal cells in acute kidney injury^38^. In addition, miR‐141 was found to be overexpressed during senescence as a result of epigenetic regulation, which may lead to reduced tissue regeneration capacity and a decline in physiological functions^39^. Some studies have argued that downregulation of miR‐141 could increase the proliferation of osteosarcoma cells and colorectal cancer SW480 cells^40^, ^41^. In addition, it has been reported that upregulating miR‐141‐3p suppresses the invasion and migration of prostate cancer cells *in vitro* 42. Those findings suggest that miR‐141 is an inhibitor of cell proliferation and osteogenic differentiation. However, the association between proliferation of BMSCs and miR‐141 still need to be investigated. In our study, we verified that miR‐141 was upregulated in SANFH and that miR‐141 could suppress the proliferation of BMSC. Our results coincided with other studies revealing that inhibition of miR‐141 could promote cell proliferation. Glucocorticoids could increase the expression of miR‐141 so that proliferation of BMSC is inhibited. Thus, we assumed that the reduction of BMSC could lead to the downregulation of osteogenic differentiation, further increasing the risk of trabecular fracture and delaying repair of bone tissues, and, finally, inducing the pathological change of SANFH.

Our research revealed that a new gene named SOX11 was associated with SANFH. SOX11 was expressed in the early passages of BMSC and decreased with replicative senescence of cells^43^. In our study, we found that SOX11 was downregulated in the group of SANFH while overexpression of SOX11 could promote the proliferation of BMSC. Our results concurred with the conclusion drawn by other researches that upregulation of SOX11 was associated with increasing proliferation of BMSC. It has been proven that blocking of glucocorticoid receptor using small interfering RNA and RU486 could upregulate the expression of SOX11 and promote the cell proliferation and capability of osteochondral differentiation in BMSC^33^, ^34^. We assumed that glucocorticoids induce cell aging, injury, and death, including apoptosis and autophagy; SOX11 may be downregulated along with the aging and death of cells. SOX11 could be a new target gene to treat and prevent SANFH due to the reason that SOX11 is correlated with proliferation and osteogenic differentiation of BMSC.

We found that miR‐141 and SOX11 were both associated with SANFH and proliferation of BMSC. We further verified that miR‐141 could directly target the expression of SOX11. Inhibition of miR‐141 upregulated the expression of SOX11 to promote the proliferation of BMSC. The association between miR‐141 and SOX11 concurred with results from separate experiments. Therefore, miR‐141 might be a positive factor to prevent or treat SANFH by targeting SOX11 and promoting proliferation. However, there are still several limitations in our study. First, we mainly concentrated on the expression of miR‐141 and SOX11 in *in vitro* study, and an *in vivo* model of SANFH should be further established and related studies should be performed to verify the regulation of miR‐141 and SOX11 in SANFH. Phosphorylation tests should also be performed. Second, we mainly focus on the proliferation of BMSC affected by miR‐141 and SOX11, and we did not perform the related experiment to investigate the differentiation of BMSC. In future research, we will further investigate the osteogenic differentiation of BMSC to verify the other roles of miR‐141 in SANFH by targeting SOX11. Third, human tissue and blood samples should be collected to further confirm the conclusions that we drew. Finally, the detail pathway involved and molecular mechanisms should be further explored.

To sum up, our results suggested that miR‐141 inhibited the proliferation of BMSC in SANFH by targeting SOX11. Inhibition of miR‐141 upregulated the expression of SOX11 and promoted the proliferation of BMSC. MiR‐141 and SOX11 could be targets to investigate the mechanism of SANFH and other related diseases, including osteoporosis and other ossification‐related diseases.

## References

[1] Tomonori S , Junichi N , Shunji K , *et al* Incidence of osteonecrosis associated with corticosteroid therapy among different underlying diseases: prospective MRI study. Rheumatology, 2011, 50: 2023–2028. 21865285 10.1093/rheumatology/ker277

[2] Vreden SG , Hermus AR , van Liessum PA , Pieters GF , Smals AG , Kloppenborg PW . Aseptic bone necrosis in patients on glucocorticoid replacement therapy. Neth J Med, 1991, 39: 153–157. 1791877

[3] Baktursun P , Hao P , Li BB . Effects of antler powder on treatment of corticosteroid‐induced avascular necrosis of the femoral head in rats. J Clin Rehabil Tissue Eng Res, 2011, 346: 1030–1031.

[4] Jones JP Jr . Fat embolism and osteonecrosis. Orthop Clin North Am, 1985, 16: 595–633. 3903602

[5] Gillet C , Dalla Valle A , Gaspard N , *et al* Osteonecrosis of the femoral head: lipotoxicity exacerbation in MSC and modifications of the bone marrow fluid. Endocrinology, 2017, 158: 490–502. 28359085 10.1210/en.2016-1687

[6] Xu X , Wen H , Hu Y , *et al* STAT1‐caspase 3 pathway in the apoptotic process associated with steroid‐induced necrosis of the femoral head. J Mol Histol, 2014, 45: 473–485. 24554068 10.1007/s10735-014-9571-6

[7] Liu W , Zhao Z , Na Y , Meng C , Wang J , Bai R . Dexamethasone‐induced production of reactive oxygen species promotes apoptosis via endoplasmic reticulum stress and autophagy in MC3T3‐E1 cells. Int J Mol Med, 2018, 41: 2028–2036. 29393368 10.3892/ijmm.2018.3412 PMC5810234

[8] Tack LJ , Tatsi C , Stratakis CA , Lodish MB . Effects of glucocorticoids on bone: what we can learn from pediatric endogenous Cushing's syndrome. Horm Metab Res, 2016, 48: 764–770. 27728929 10.1055/s-0042-117721

[9] Ohnaka K , Tanabe M , Kawate H , Nawata H , Takayanagi R . Glucocorticoid suppresses the canonical Wnt signal in cultured human osteoblasts. Biochem Biophys Res Commun, 2005, 329: 177–181. 15721290 10.1016/j.bbrc.2005.01.117

[10] Wu Z , Bucher NL , Farmer SR . Induction of peroxisome proliferator‐activated receptor gamma during the conversion of 3T3 fibroblasts into adipocytes is mediated by C/EBPbeta, C/EBPdelta, and glucocorticoids. Mol Cell Biol, 1996, 16: 4128–4136. 8754811 10.1128/mcb.16.8.4128 PMC231409

[11] Friedenstein AJ , Petrakova KV , Kurolesova AI , Frolova GP . Heterotopic of bone marrow. Analysis of precursor cells for osteogenic and hematopoietic tissues. Transplantation, 1968, 6: 230–247. 5654088

[12] Matsuya H , Kushida T , Asada T , Umeda M , Wada T , Iida H . Regenerative effects of transplanting autologous mesenchymal stem cells on corticosteroid‐induced osteonecrosis in rabbits. Mod Rheumatol, 2008, 18: 132–139. 18288561 10.1007/s10165-008-0023-6

[13] Pittenger MF , Mackay AM , Beck SC , *et al* Multilineage potential of adult human mesenchymal stem cells. Science, 1999, 284: 143–147. 10102814 10.1126/science.284.5411.143

[14] Prockop DJ . Marrow stromal cells as stem cells for nonhematopoietic tissues. Science, 1997, 276: 71–74. 9082988 10.1126/science.276.5309.71

[15] Tian L , Yu X . Lipid metabolism disorders and bone dysfunction‐interrelated and mutually regulated (review). Mol Med Rep, 2015, 12: 783–794. 25760577 10.3892/mmr.2015.3472 PMC4438959

[16] Xiang S , Li Z , Weng X . The role of lncRNA RP11‐154D6 in steroid‐induced osteonecrosis of the femoral head through BMSC regulation. J Cell Biochem, 2019, 120: 18435–18445. 31190361 10.1002/jcb.29161

[17] Zhong LN , Zhang YZ , Li H , Fu HL , Lv CX , Jia XJ . Overexpressed miR‐196a accelerates osteogenic differentiation in osteoporotic mice via GNAS‐dependent hedgehog signaling pathway. J Cell Biochem, 2019, 120(12): 19422–19431. 31452264 10.1002/jcb.29166

[18] Contador D , Ezquer F , Espinosa M , *et al* Featured article: dexamethasone and rosiglitazone are sufficient and necessary for producing functional adipocytes from mesenchymal stem cells. Exp Biol Med, 2015, 240: 1235–1246. 10.1177/1535370214566565 PMC4935363 25595190

[19] Yao W , Dai W , Jiang L , *et al* Sclerostin‐antibody treatment of glucocorticoid‐induced osteoporosis maintained bone mass and strength. Osteoporos Int, 2016, 27: 283–294. 26384674 10.1007/s00198-015-3308-6 PMC4958115

[20] Lee JS , Lee JS , Roh HL , Kim CH , Jung JS , Suh KT . Alterations in the differentiation ability of mesenchymal stem cells in patients with nontraumatic osteonecrosis of the femoral head: comparative analysis according to the risk factor. J Orthop Res, 2006, 24: 604–609. 16514658 10.1002/jor.20078

[21] Lee RC , Feinbaum RL , Ambros V . The *C. elegans* heterochronic gene lin‐4 encodes small RNAs with antisense complementarity to lin‐14. Cell, 1993, 75: 843–854. 8252621 10.1016/0092-8674(93)90529-y

[22] Bentwich I , Avniel A , Karov Y , *et al* Identification of hundreds of conserved and nonconserved human microRNAs. Nat Genet, 2005, 37: 766–770. 15965474 10.1038/ng1590

[23] Farazi TA , Spitzer JI , Morozov P , Tuschl T . miRNAs in human cancer. J Pathol, 2015, 223: 102–115. 10.1002/path.2806 PMC3069496 21125669

[24] He L , Hannon GJ . MicroRNAs: small RNAs with a big role in gene regulation. Nat Rev Genet, 2004, 5: 522–531. 15211354 10.1038/nrg1379

[25] Shi C , Huang P , Kang H , *et al* Glucocorticoid inhibits cell proliferation in differentiating osteoblasts by microRNA‐199a targeting of WNT signaling. J Mol Endocrinol, 2015, 54: 325–337. 25878056 10.1530/JME-14-0314

[26] Li H , Li T , Fan J , *et al* miR‐216a rescues dexamethasone suppression of osteogenesis, promotes osteoblast differentiation and enhances bone formation, by regulating c‐Cbl‐mediated PI3K/AKT pathway. Cell Death Differ, 2015, 22: 1935–1945. 26206089 10.1038/cdd.2015.99 PMC4816120

[27] Bian Y , Qian W , Hongling LI , Zhao RC , Shan WX , Weng X . Pathogenesis of glucocorticoid‐induced avascular necrosis: a microarray analysis of gene expressionin vitro. Int J Mol Med, 2015, 36: 678–684. 26151338 10.3892/ijmm.2015.2273 PMC4533777

[28] Finlay‐Schultz J , Cittelly DM , Hendricks P , *et al* Progesterone downregulation of miR‐141 contributes to expansion of stem‐like breast cancer cells through maintenance of progesterone receptor and Stat5a. Oncogene, 2015, 34: 3676–3687. 25241899 10.1038/onc.2014.298 PMC4369481

[29] Sangani R , Periyasamy‐Thandavan S , Kolhe R , *et al* MicroRNAs‐141 and 200a regulate the SVCT2 transporter in bone marrow stromal cells. Mol Cell Endocrinol, 2015, 410: 19–26. 25617715 10.1016/j.mce.2015.01.007 PMC4824062

[30] Jethon A , Pula B , Olbromski M , *et al* Prognostic significance of SOX18 expression in non‐small cell lung cancer. Int J Oncol, 2015, 46: 123–132. 25310193 10.3892/ijo.2014.2698

[31] Wang L , Shen YF , Shi ZM , Shang XJ , Jin DL , Xi F . Overexpression miR‐211‐5p hinders the proliferation, migration, and invasion of thyroid tumor cells by downregulating SOX11. J Clin Lab Anal, 2017, 32: e22293. 10.1002/jcla.22293 PMC6817049 28703321

[32] Fang G , Liu J , Wang Q , *et al* MicroRNA‐223‐3p regulates ovarian cancer cell proliferation and invasion by targeting SOX11 expression. Int J Mol Sci, 2017, 18: 1208. 10.3390/ijms18061208 PMC5486031 28587313

[33] Hong L , Wei N , Joshi V , *et al* Effects of glucocorticoid receptor small interfering RNA delivered using poly lactic‐co‐glycolic acid microparticles on proliferation and differentiation capabilities of human mesenchymal stromal cells. Tissue Eng Part A, 2012, 18: 775–784. 21988716 10.1089/ten.tea.2011.0432 PMC3313605

[34] Yu Y , Wei N , Stanford C , Schmidt T , Hong L . In vitro effects of RU486 on proliferation and differentiation capabilities of human bone marrow mesenchymal stromal cells. Steroids, 2012, 77: 132–137. 22093480 10.1016/j.steroids.2011.10.017 PMC3242919

[35] Zhang L , Sun X , Tian D , *et al* Model establishment, MRI and pathological features of early steroid‐induced avascular necrosis of femoral head in rabbit. Zhongguo Xiu Fu Chong Jian Wai Ke Za Zhi, 2015, 29: 1240–1243. 26749731

[36] Bai R , Feng W , Liu WL , *et al* Roles of osteocyte apoptosis in steroid‐induced avascular necrosis of the femoral head. Genet Mol Res, 2016, 15(1). 10.4238/gmr.15017529. 27050956

[37] Wang T , Teng S , Zhang Y , Wang F , Ding H , Guo L . Role of mesenchymal stem cells on differentiation in steroid‐induced avascular necrosis of the femoral head. Exp Ther Med, 2017, 13: 669–675. 28352349 10.3892/etm.2016.3991 PMC5348717

[38] de Almeida DC , Bassi EJ , Azevedo H , *et al* A regulatory miRNA‐mRNA network is associated with tissue repair induced by mesenchymal stromal cells in acute kidney injury. Front Immunol, 2016, 7: 645. 28096802 10.3389/fimmu.2016.00645 PMC5206861

[39] Yu KR , Lee S , Jung JW , *et al* MicroRNA‐141‐3p plays a role in human mesenchymal stem cell aging by directly targeting ZMPSTE24. J Cell Sci, 2013, 126: 5422–5431. 24101728 10.1242/jcs.133314

[40] Wang N , Li P , Liu W , *et al* miR‐141‐3p suppresses proliferation and promotes apoptosis by targeting GLI2 in osteosarcoma cells. Oncol Rep, 2018, 39: 747–754. 29251328 10.3892/or.2017.6150

[41] Long ZH , Bai ZG , Song JN , *et al* miR‐141 inhibits proliferation and migration of colorectal cancer SW480 cells. Anticancer Res, 2017, 37: 4345–4352. 28739727 10.21873/anticanres.11828

[42] Huang S , Wa Q , Pan J , *et al* Downregulation of miR‐141‐3p promotes bone metastasis via activating NF‐kappaB signaling in prostate cancer. J Exp Clin Cancer Res, 2017, 36: 173. 29202848 10.1186/s13046-017-0645-7 PMC5716366

[43] Larson BL , Ylostalo J , Lee RH , Gregory C , Prockop DJ . Sox11 is expressed in early progenitor human multipotent stromal cells and decreases with extensive expansion of the cells. Tissue Eng Part A, 2010, 16: 3385–3394. 20626275 10.1089/ten.tea.2010.0085 PMC2965191

